# *Pseudomonas* 2.0: genetic upgrading of *P. putida* KT2440 as an enhanced host for heterologous gene expression

**DOI:** 10.1186/s12934-014-0159-3

**Published:** 2014-11-11

**Authors:** Esteban Martínez-García, Pablo I Nikel, Tomás Aparicio, Víctor de Lorenzo

**Affiliations:** Systems and Synthetic Biology Program, Centro Nacional de Biotecnología (CNB-CSIC), Campus de Cantoblanco, 28049 Madrid, Spain

**Keywords:** *Pseudomonas putida*, Heterologous gene expression, *Chassis*, Reducing power, Stress resistance, Metabolic robustness, Flagellum, Metabolic engineering

## Abstract

**Background:**

Because of its adaptability to sites polluted with toxic chemicals, the model soil bacterium *Pseudomonas putida* is naturally endowed with a number of metabolic and stress-endurance qualities which have considerable value for hosting energy-demanding and redox reactions thereof. The growing body of knowledge on *P. putida* strain KT2440 has been exploited for the rational design of a derivative strain in which the genome has been heavily edited in order to construct a robust microbial cell factory.

**Results:**

Eleven non-adjacent genomic deletions, which span 300 genes (i.e., 4.3% of the entire *P. putida* KT2440 genome), were eliminated; thereby enhancing desirable traits and eliminating attributes which are detrimental in an expression host. Since ATP and NAD(P)H availability – as well as genetic instability, are generally considered to be major bottlenecks for the performance of platform strains, a suite of functions that drain high-energy phosphate from the cells and/or consume NAD(P)H were targeted in particular, the whole flagellar machinery. Four prophages, two transposons, and three components of DNA restriction-modification systems were eliminated as well. The resulting strain (*P. putida* EM383) displayed growth properties (i.e., lag times, biomass yield, and specific growth rates) clearly superior to the precursor wild-type strain KT2440. Furthermore, it tolerated endogenous oxidative stress, acquired and replicated exogenous DNA, and survived better in stationary phase. The performance of a bi-cistronic GFP-LuxCDABE reporter system as a proxy of combined metabolic vitality, revealed that the deletions in *P. putida* strain EM383 brought about an increase of >50% in the overall physiological vigour.

**Conclusion:**

The rationally modified *P. putida* strain allowed for the better functional expression of implanted genes by directly improving the metabolic currency that sustains the gene expression flow, instead of resorting to the classical genetic approaches (e.g., increasing the promoter strength in the DNA constructs of interest).

**Electronic supplementary material:**

The online version of this article (doi:10.1186/s12934-014-0159-3) contains supplementary material, which is available to authorized users.

## Introduction

Since the onset of the recombinant DNA era, heterologous gene expression has been one of the pillars of contemporary Metabolic Engineering [1]. The implicit assumption is that DNA acts as a sort of *software* which, if entered in a *reading machine* already in place (the host), will result in the expression of the genes at stake at the user’s will [2,3]. This somewhat naïve concept has proven, however, very successful, and the number of genes and pathways that have been functionally expressed in archetypal hosts such as *Escherichia coli* just by knocking-in the DNA sequences of interest is very large [4–6]. This view has been exacerbated in the recent times with the inception of Synthetic Biology, which entertains the performance of a biological *chassis* (i.e., the basic, complete genetic, and biochemical scaffold needed for the gene expression flow [7]) in which different engineered DNA constructs are plugged-in and out for specific purposes. Such scenario, however, is often hampered by a large number of constrains that the host imposes on the efficiency of the gene expression process. These hurdles include (but are not limited to) [i] the toxicity of certain amino acid sequences that fold poorly and saturate the chaperoning ability of the host cells [8,9], [ii] the stress caused by the encoded biological activities (e.g., enzymes and their metabolic products) on the endogenous biochemical network [10,11], and [iii] the drain of metabolic currency that is diverted into production of the implanted gene(s) and/or pathway(s), a phenomenon termed *metabolic burden* [12,13]. In this context, it seems somewhat paradoxical that most efforts for improving heterologous expression of biological functions have focused on refining the DNA sequence of the implant (e.g., codon usage, promoter strength, alternative ribosome binding sites, and engineering of the intergenic regions [4,5,14–16]), and very few attempts questioned which could be the optimal host for specific purposes. While multiple directed deletions in the extant genome of *E. coli* [17–19] resulted in a clearly improved microbial cell factory and a more stable carrier of foreign genes, the background metabolism and the built-in ability to endure stress remain exactly as those of an enteric bacterium – which is not habituated to host harsh reactions that are common in industrial biotechnology and biocatalysis [11,20].

Fortunately, naturally-occurring environmental microorganisms have already dealt with the evolutionary challenge of acquiring and expressing new genes and metabolic pathways for very toxic substrates. A most typical scenario is that of mobile catabolic plasmids, that spread through microbial consortia in sites polluted with industrial wastes. It is not casual that the species that host such plasmids (frequently encoding several oxidative biotransformations of complex organic compounds) do not belong to Enterobacteriaceae, but they are often members of the genus *Pseudomonas* [21–23]. One of the reasons for this occurrence is the vigorous Entner-Doudoroff and pentose phosphate pathways present in most *Pseudomonas* species, thus resulting in high rates of NADPH regeneration, which in turn helps counteracting both endogenous and exogenous oxidative stress [24,25]. This trait provides the right metabolic frame for running enzymatic pathways that other bacterial would be unable to cope with.

On these bases, it does not come as a surprise that *P. putida* KT2440, a non-pathogenic strain of the soil bacterium *P. putida* is being increasingly used as a host for heterologous DNA expression for different biotechnological purposes [26–28]. This strain is not only certified as GRAS (*g*enerally *r*egarded *a*s *s*afe [29]) and endowed with a remarkable metabolic versatility, but it also possesses a noteworthy tolerance to many organic compounds [28,30] and other stressful conditions, such as those that generate reactive oxygen species (ROS). Still, the intrinsic value of this strain for bearing heterologous genes is flawed by the innate diversion of metabolic currency [in particular ATP and NAD(P)H] into biological functions that are useful under natural conditions but altogether useless in an industrial bioprocess [31].

In this work, the genome of *P. putida* KT2440 was inspected to identify regions conspicuously liable of limiting heterologous gene expression – whether because they are associated to genetic instability or owing to the non-productive consumption of metabolic resources. The targeted deletion of 11 chromosomal regions (comprising 300 genes) of this bacterium is shown below to result in *P. putida* variants equipped with an enhanced ability to host artificially implanted genes. In particular, the simultaneous deletion of the complete proviral load and the whole flagellar machinery upgraded very significantly every descriptor of physiological performance observed in the naturally occurring host. These results expose how the metabolic frame sustaining the gene expression flow can be rationally streamlined for the sake of a better functionality of the cognate platform strain.

## Results and discussion

### Identifying the bottlenecks of *P. putida* KT2440 as a functional host of foreign genes

**Figure 1:**
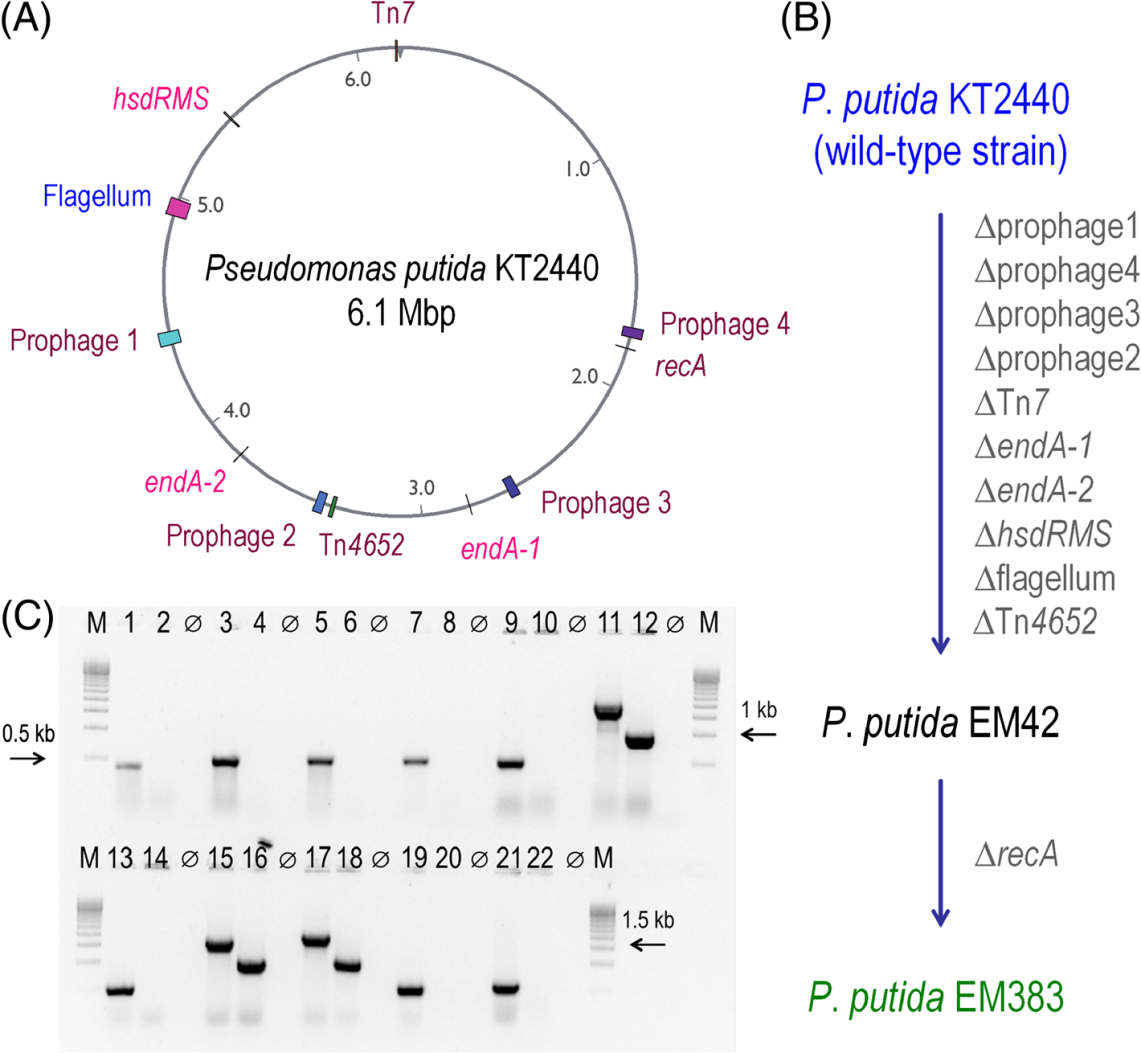
**Operons and genomic regions deleted in** ***P*** **.** ***putida*** **KT2440 to construct a cell factory strain. (A)** Position of the eleven gene(s)/regions deleted in wild-type *P. putida* KT2440 indicated in the physical map of the chromosome. **(B)** Roadmap for the construction of strains EM42 and EM383. Relevant genes are depicted in the order in which they were eliminated (see also Additional file 1: Table S1). **(C)** Electrophoresis of the diagnostic PCR amplifications to confirm the deletions. The flanking lanes (M) correspond to a DNA ladder [500-bp Molecular Ruler *EZ* Load™ (Bio-Rad Corp., Berkeley, CA, USA)], and lanes identified as ϕ are negative controls, i.e., samples without DNA template. The photograph shows the products resulting from PCR amplifications of [i] an internal gene within prophage 1, KT2440 (lane 1) and EM383 (lane 2); [ii] an internal gene of prophage 2, KT2440 (lane 3) and EM383 (lane 4); [iii] an internal gene of prophage 3, KT2440 (lane 5) and EM383 (lane 6); [iv] an internal gene of prophage 4, KT2440 (lane 7) and EM383 (lane 8); [v] an internal gene of the *hsdRMS* operon, KT2440 (lane 9) and EM383 (lane 10); [vi] the TS1-TS2 region of *recA*, KT2440 (lane 11) and EM383 (lane 12); [vii] an internal gene of the Tn*7*-like operon, KT2440 (lane 13) and EM383 (lane 14); [viii] the TS1-TS2 region of *endA-1*, KT2440 (lane 15) and EM383 (lane 16); [ix] the TS1-TS2 region of *endA-2*, KT2440 (lane 17) and EM383 (lane 18); [x] an internal gene of the flagellar operon, KT2440 (lane 19) and EM383 (lane 20); and [xi] an internal gene of the Tn*4652* operon, KT2440 (lane 21) and EM383 (lane 22). The details of primers sequence used in these amplifications are given in Additional file 1: Table S2.

This study capitalizes on the intrinsic physiological and metabolic strength of *P. putida* KT2440 in the quest for an improved host of heterologous gene expression. One major constraint for such process is ensuring sufficient ATP availability to fuel the action of GroEL/ES in folding foreign polypeptides, which are often produced at high levels by the strong promoters of typical recombinant expression systems [32,33]. In fact, GroEL/ES seems to be the cell component that most avidly hydrolyzes ATP [34]. On the other hand, metabolic stress, which can cause ROS formation, is often accompanied by a higher consumption of reducing power [i.e., NAD(P)H] [10,22]. This situation indicates that engineering increased intracellular ATP and/or NAD(P)H levels is predicted to result in a better expression host. On the other hand, the implantation and performance of recombinant constructs is exposed to the many chromosomal elements that cause genetic instability and rejection of foreign genes, e.g., insertion sequences (IS), transposons, prophages, and DNA restriction systems. On this basis, the annotated genomic sequence of strain KT2440 (available on line in the *Pseudomonas* Genome Database [35]) was inspected to spot DNA segments encoding tasks which, while being non-essential, either grossly drain much metabolic currency or are likely to cause genomic instability. A tentative survey of such segments yield a minimum of 11 chromosomal sites determining a variety of functions (Figure 1A), the removal of which is justified as follows. First, there is a whole of 4 non-contiguous large segments (~170 kb in total, representing 2.6% of the genome of strain KT2440), encoding prophages known to display various degrees of activity [36]. These sequences are genuinely parasitic, and they make cells more sensitive to DNA damage and, when induced, they cause stochastic lysis in the bacterial population. Then it comes the 54 ISs (called ISPpu) and other mobile DNA elements borne by *P. putida,* which account for ~1% of the genome of *P. putida* KT2440 [37,38], and which are poised to counterselect knocked-in constructs that may burden the host [39,40]. While targeting all of them individually is beyond the scope of this work, two conspicuous cases were addressed. One instance is the 15.7-kb Tn*4652* transposon [37,41], a member of the Tn*3* transposon family which spans the open reading frames (ORFs) PP2964-PP2984 in the genome. Why is it relevant to focus on this transposon? While other mobile elements of the *P. putida* KT2440 are surely functional, Tn*4652* is the only case in which its *in vivo* activity has been well accredited so far, especially when cells face C starvation [42–44]. A second genomic segment with the potential to cause instability of recombinant constructs (especially those assembled in Tn*7* transposon vectors) spans ORFs PP5404-PP5407, and encodes a complete Tn*7*-like transposase cluster [37,41]. This genetic locus may interfere with inserts targeted at the specific Tn*7* attachment site of the *P. putida* chromosome that is often used for stable introduction of foreign DNA [45,46], and was targeted as well as a potential cause of genetic instability.

Another type of undesirable traits are those that affect the physical integrity of incoming DNA. The genome of *P. putida* KT2440 bears two genes, termed *endA-1* and *endA-2*, encoding two deoxyribonucleases I (i.e., type I DNases) that degrade double-stranded DNA in a sequence-independent fashion [47]. EndA-2 is predicted to be in the periplasm (like in the case of the homologous EndA of *E. coli*), while EndA-1 could also be released extracellularly [35]. These enzymes both nick exogenously added plasmids and are known to degrade plasmid DNA extracted from cells which have DNase I activity [48]. The presence of two such enzymes in *P. putida* surely adds to the difficulty to both enter and retrieve plasmids [49,50]. In addition, strain KT2440 has an *hsdRMS* operon (PP4740-PP4742) encoding a type I DNA restriction-modification system, which typically protect the bacterium against foreign DNA while facilitating the recombination between the bacterial genome and the newly incoming DNA [51]. Although *P. putida* KT2440 was first described as a naturally occurring *hsdR1* strain [52], the complete (and possibly functional) *hsdRMS* genes was entered in the list of chromosomal segments to be removed. In contrast, no evidence of additional mechanisms of active *in vivo* degradation of incoming DNA was found*,* whether systems based on clustered regularly interspaced short palindromic repeats (CRISP [53]) or the bacterial Argonaute complexes [54]. Finally, although recombination between incoming DNA and endogenous genomic sequences is not very efficient in *P. putida* KT2440 [55], the option of removing *recA* was also considered to further decrease chances of unpredictable genetic changes [56].

The genomic sites identified thus far were candidates to flaw the ease of handling and the stability of engineered constructs in *P. putida*. Yet, they were still alien to the problem raised above regarding the waste of metabolic currency caused by expression of recombinant genes or metabolic pathways. In this case, it is plausible that the second more important cause of energy consumption (besides the afore-mentioned GroES/EL machinery) is the motion of the flagellar rotor. Removal of the flagellum of *P. putida* has been recently shown to result in cells with a higher capacity to endure environmental stresses [57]. This feature was accompanied by a net increase of intracellular ATP and NADPH, as well as a considerable enhancement in the energy charge and redox ratios [57]. Since lacking flagella would not be overly disadvantageous in shaken flasks or in a bioreactor, one could think on diverting the surplus of ATP and reducing power into the improvement of heterologous gene expression. On this basis, the complete flagellar operon of *P. putida* KT2440 (ORFs PP4329-PP4397, stretching for ~69 kb [57]) was included in the list of genomic sites to be deleted in addition to those related to genetic instability mentioned before.

### Construction of the streamlined cell-factory strains *P. putida* EM42 and *P. putida* EM383

The rationale above was translated into the sequential deletion of all the DNA segments shown in Figure 1A by using the procedure developed by Martínez-García *et al*. [58,59]. The method mediates the seamless excision of genomic DNA segments of variable sizes with virtually no acquisition of additional mutations. The starting point of the deletion flowchart was the derivative of *P. putida* KT2440 deleted of the 4 prophage elements, named *P. putida* ∆all-Φ [36]. The subsequent removal of selected segments was completed by following the order ∆all-Φ → ∆Tn*7*-like transposase → ∆*endA-1* → ∆*endA-2* → ∆*hsdRMS* → ∆flagellum → ∆Tn*4652* (Figure  1B and Table 1), thereby resulting in what was called cell-factory strain *P. putida* EM42. A further deletion of the *recA* gene (Figure 1B and Table 1) was then introduced in *P. putida* EM42 to originate a second cell-factory variant, named *P. putida* EM383. This deletion has obviously to be the last one as it impedes any further recombination on which the genome editing procedure is based. The precise extension and the coordinates of each of the 11 deletions can be found in Additional file 1: Table S1. The predicted boundaries of each of them were verified by amplifying and sequencing the corresponding flanking segments, and their maintenance was followed at each round of excisions through diagnostic PCR amplification of different portions of the target regions (Figure 1C). Whenever possible, deletions were limited to the start and the end of the genes of interest. However, elimination of the flagellar operon (PP4329 to PP4397) also removed the last four bases of ORF PP4328 (encoding an hypothetical protein of unknown function), as the target genes overlap with the start of ORF PP4329 [57]. In total, the whole process excised 300 genes, that represent 4.3% of the genome of the parental *P. putida* strain.

**Table 1:** **Bacterial strains and plasmids used in this work**

**Strain or plasmid**	**Relevant characteristics** ^***a***^	**Reference or source**
*Escherichia coli*		
DH5α	Cloning host; F^−^ λ^−^ *endA1 glnX44*(AS) *thiE1 recA1 relA1 spoT1 gyrA96*(Nal^R^) *rfbC1 deoR nupG* Φ80(*lacZ*Δ*M15*) Δ(*argF*-*lac*)*U169 hsdR17*(*r*_*K*_^−^ *m*_*K*_^*+*^)	[60]
DH5α λ*pir*	Cloning host; λ*pir* lysogen of strain DH5α	[61]
HB101	Helper strain; F^−^ λ^−^ *hsdS20*(*r*_*B*_^−^ *m*_*B*_^−^) *recA13 leuB6*(Am) *araC14* Δ(*gpt-proA*)*62 lacY1 galK2*(Oc) *xyl-5 mtl-1 thiE1 rpsL20*(Sm^R^) *glnX44*(AS)	[62]
*Pseudomonas putida*		
KT2440	Wild-type strain; mt-2 derivative cured of the TOL plasmid pWW0	[52]
KT2440 Δall-Φ	KT2440 derivative; Δprophage1 Δprophage4 Δprophage3 Δprophage2	[36]
EM42	KT2440 derivative; Δprophage1 Δprophage4 Δprophage3 Δprophage2 ΔTn*7* Δ*endA-1* Δ*endA-2* Δ*hsdRMS* Δflagellum ΔTn*4652*	This work
EM383	KT2440 derivative; EM42 Δ*recA*	This work
Plasmids		
pRK600	Helper plasmid used for conjugation; *oriV*(ColE1), RK2(*mob* ^*+*^ *tra* ^*+*^); Cm^R^	[63]
pEMG	Plasmid used for deletions; *oriV*(R6K), *lacZ*α fragment with two flanking I-*Sce*I recognition sites; Km^R^	[58]
pSW-I	Helper plasmid used for deletions; *oriV*(RK2), *xylS*, *Pm→I-Sce*I; Ap^R^	[64]
pEMG-Tn*7*	pEMG bearing an 1.6-kb TS1-TS2 *Eco*RI-*Xma*I insert for deleting the PP5404-PP5407 operon	This work
pEMG-*endA-1*	pEMG bearing an 1-kb TS1-TS2 *Xma*I-*Bam*HI insert for deleting the *endA-1* gene	This work
pEMG-*endA-2*	pEMG bearing an 1-kb TS1-TS2 *Eco*RI-*Bam*HI insert for deleting the *endA-2* gene	This work
pEMG-*hsdRMS*	pEMG bearing an 1.3-kb TS1-TS2 *Eco*RI-BamHI insert for deleting the *hsdRMS* operon	This work
pEMG-flagella	pEMG bearing an 1.5-kb TS1-TS2 *Eco*RI-*Bam*HI insert for deleting the flagellar operon	[57]
pEMG-Tn*4652*	pEMG bearing an 1-kb TS1-TS2 *Xma*I-*Bam*HI insert for deleting the Tn*4652* transposon	This work
pEMG-*recA*	pEMG bearing an 1-kb TS1-TS2 *Eco*RI-*Bam*HI insert for deleting the *recA* gene	[36]
pSEVA221	Cloning vector; *oriV*(RK2); standard multiple cloning site; Km^R^	[65]
pSEVA251	Cloning vector; *oriV*(RFS1010); standard multiple cloning site; Km^R^	[65]
pGL-XP	Expression plasmid; *oriV*(pBBR1), *oriT*, *xylS*, *Pm→gfp-luxCDABE*; Km^R^ Sm^R^	Benedetti *et al*., in preparation

^*a*^Antibiotic markers: Ap, ampicillin; Cm, chloramphenicol; Km, kanamycin; Nal, nalidixic acid; Sm, streptomycin.

The new physiological and genetic properties that surfaced in the multi-deleted strain *P. putida* EM383 are described in the following sections. Note that, instead of discussing the independent contributions of each of the eliminated genes to the observed phenotypes, all of them are considered to be the result of a block intervention in the extant genome of *P. putida* KT2440 for the sake of upgrading its performance as a host for heterologous gene expression.

### Gross physiological properties of the cell-factory strain *P. putida* EM383

**Figure 2:**
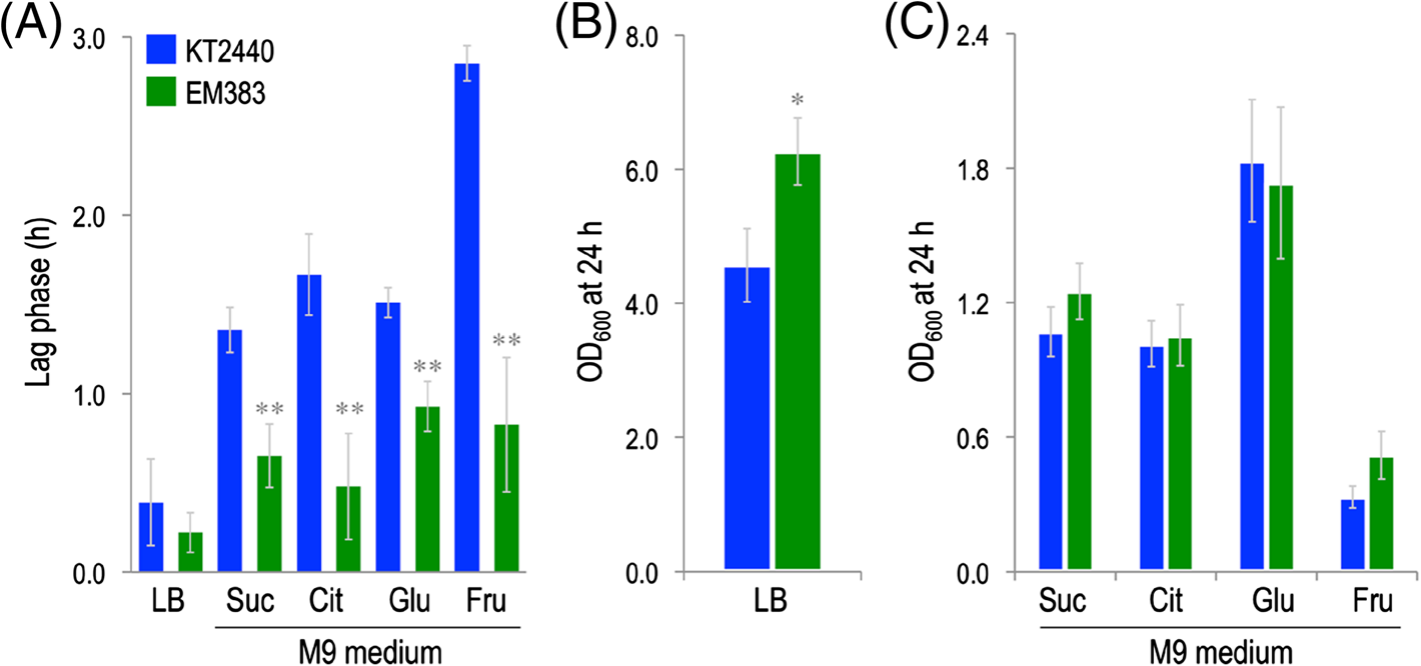
**Growth parameters of** ***P*** **.** ***putida*** **KT2440 and the streamlined strain EM383. (A)** Duration of the lag phase of wild-type KT2440 cells (blue) and the streamlined strain EM383 (green) in rich LB medium or M9 minimal medium added with 0.2% (w/v) of either succinate (Suc), citrate (Cit), glucose (Glu), or fructose (Fru). The extent of the lag phase was calculated using data from growth curves as described by Dalgaard and Koutsoumanis [67]. **(B)** Final cell density (estimated as the optical density at 600 nm, OD_600_) of shaken-flask cultures of wild-type KT2440 (blue) and the streamlined strain EM383 (green) in rich LB medium. **(C)** Final cell density (estimated as the OD_600_) of shaken-flask cultures of wild-type KT2440 (blue) and the streamlined strain EM383 (green) in M9 minimal medium added with 0.2% (w/v) of either succinate (Suc), citrate (Cit), glucose (Glu), or fructose (Fru). In all cases, the mean values of the corresponding parameter are plotted along with the SD of three independent experiments. The asterisks indicate a significant difference in the corresponding parameter when comparing strain EM383 and wild-type KT2440 according to the Student’s *t* test (*, *P* <0.05; and **, *P* <0.01).

The first observable traits acquired by the multi-deleted strain *P. putida* EM383 were revealed by comparing its growth properties with those of the wild-type KT2440 strain. These tests were made both in rich LB medium and in M9 minimal medium supplemented with C sources that elicit different metabolic regimes, i.e., succinate and citrate for gluconeogenesis, or glucose and fructose for glycolysis. Three separate growth parameters were considered to this end. First, the duration of the lag phase before cells take off to grow exponentially was assessed. This parameter seems to be associated with the ability of cells to overcome the oxidative damage that is inherited from the stationary phase that they come from [66]. To examine this issue in our strains, growth curves were carried out in 96-well microtiter plates inoculated with an standard number of cells of each strain coming from overnight cultures, and then passed to the different culture media as explained in the Methods section. Inspection of the data of Figure 2A revealed that *P. putida* EM383 had a significantly shorter lag phase than wild-type cells in all instances, the effect being more evident when fructose was used as C source (2.8 ± 0.1 h for the wild-type strain *vs*. 0.8 ± 0.3 h for the EM383 streamlined strain). Since this early take off was observed before in *P. putida* cells lacking the flagella, it is likely that the property acquired by strain EM383 is due to the loss of the same genes (see below). The shorter lag phase phenomenon makes sense as cells without the flagellar operon have an increased level of NADPH that probably helps mitigating oxidative stress [57].

**Figure 3:**
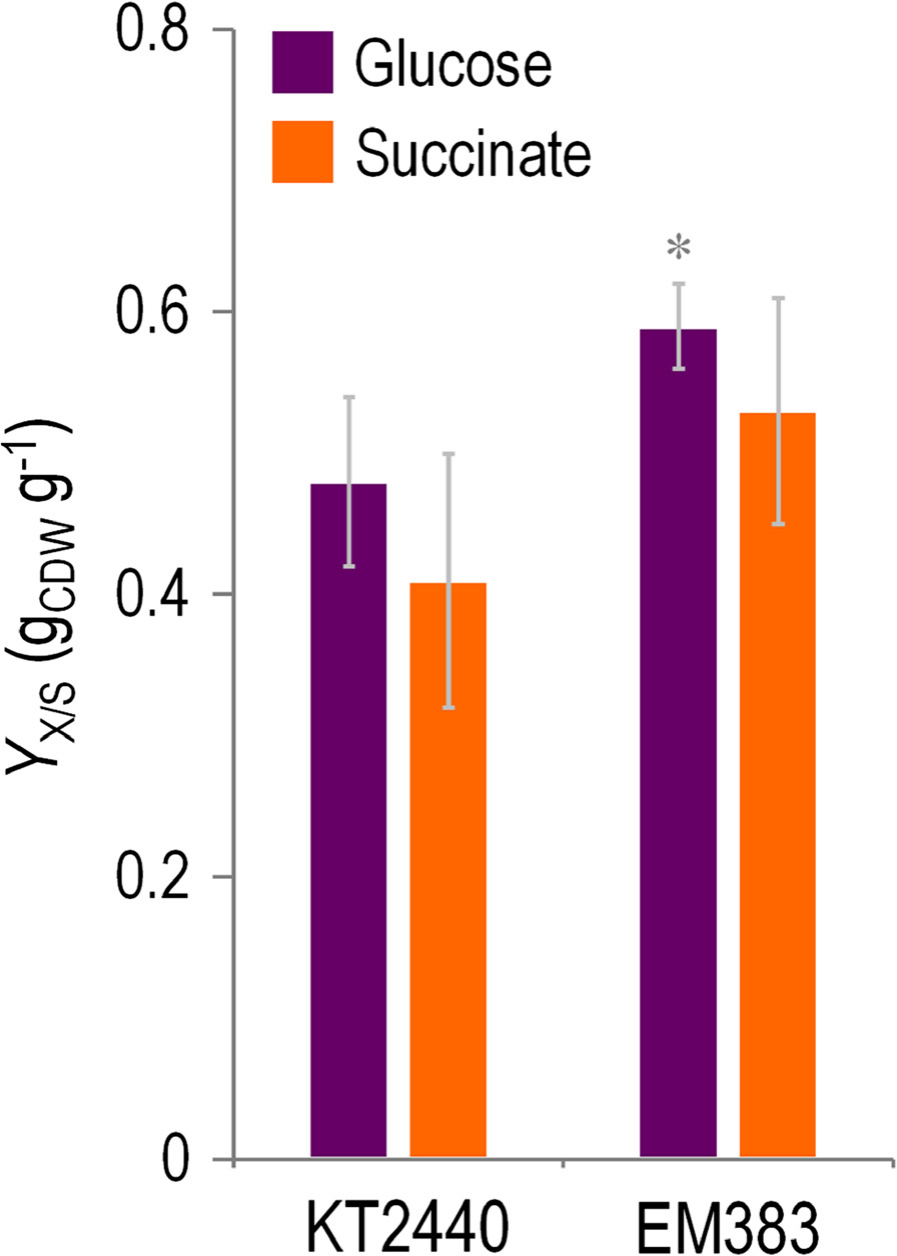
**Biomass yields of wild-type** ***P*** **.** ***putida*** **KT2440 and the streamlined strain EM383 in shaken-flask cultures.** Biomass yield coefficients (*Y*_X/S_) of wild-type *P. putida* KT2440 and *P. putida* EM383 were estimated in glucose and succinate shaken-flask cultures, as representative glycolytic and gluconeogenic C sources, respectively. Cells were grown as described in the Methods section, and the biomass yield coefficients were calculated during exponential growth by measuring the formation of biomass (CDW, cell dry weight) and the specific rate of substrate consumption. Growth parameters were based on three independent biological experiments conducted in triplicates, and the bars represent the mean value of the corresponding parameter and SD. The asterisk (*) indicates a significant difference for strain EM383 as compared to wild-type KT2440 according to the Student’s *t* test (*P* <0.05).

The second important physiological parameter was the maximum growth rate. As shown in Table 2, the differences between strains in this case were not significant, except for LB cultures, where growth of the wild-type strain was slightly better. In all, these figures indicate that the multiple deletions introduced do not significantly affect the growth performance of strain EM383. However, it is to notice that faster growth also means more oxidative stress [68,69], which needs to be counteracted to the detriment of the NAD(P)H pool [70], thereby resulting in a reduced biomass yield. This prediction was confirmed when the final optical density at 600 nm (OD_600_) was assessed in shaken-flask cultures following 24 h of vigorous shaking (Figure 2B and C). Under these conditions, *P. putida* EM383 reached OD_600_ values both in LB medium (Figure 2B) and in M9 minimal medium amended with fructose (Figure 2C) that was remarkably higher than those for the wild-type strain. Note that fructose is the only C source that can be diverted through a standard glycolytic route in *P. putida* KT2440 [71]. These differences were exacerbated when the cultures were subject to a more intense aeration (data not shown), suggesting that the observed effect is connected to the way either strain deals with oxidative stress and ROS formation. Should that be the case, differences between the strains had to be noticed in cultures with glucose and succinate, which do not manifest in the data of Figure 2C. However, when the biomass yield coefficients (*Y*_X/S_) of the wild-type *P. putida* KT2440 and *P. putida* EM383 on glucose and succinate cultures (Figure 3) were accurately calculated, a significant divergence between the two was observed in favour of the multi-deleted strain. Every growth parameter thus accredits a gross gain of physiological performance in *P. putida* EM383 cells.

**Table 2:** **Growth characterization of the wild-type** ***P*** **.** ***putida*** **KT2440 and the streamlined strain EM383 using different C sources**

***P*** **.** ***putida*** **strain**	**Specific growth rate** ^***a***^ **(h** ^**−1**^ **) in:**				
	**LB medium**	**M9 minimal medium amended with**			
		**Succinate**	**Citrate**	**Glucose**	**Fructose**
KT2440	0.74 ± 0.01	0.44 ± 0.05	0.46 ± 0.08	0.32 ± 0.02	0.20 ± 0.01
EM383	0.56 ± 0.08	0.41 ± 0.05	0.39 ± 0.04	0.40 ± 0.1	0.22 ± 0.01

^*a*^The specific growth rate for each strain was calculated during exponential growth. The cultures were carried out in 96-well microtiter plates, and the optical density at 600 nm was measured every 15 min during 24 h using a SpectraMax M2e microplate reader. All tested C sources were added to M9 minimal medium at 0.2% (w/v). Results represent the mean and SD of three independent experiments.

### Metabolic descriptors of the streamlined cell-factory *P. putida* strain

**Figure 4:**
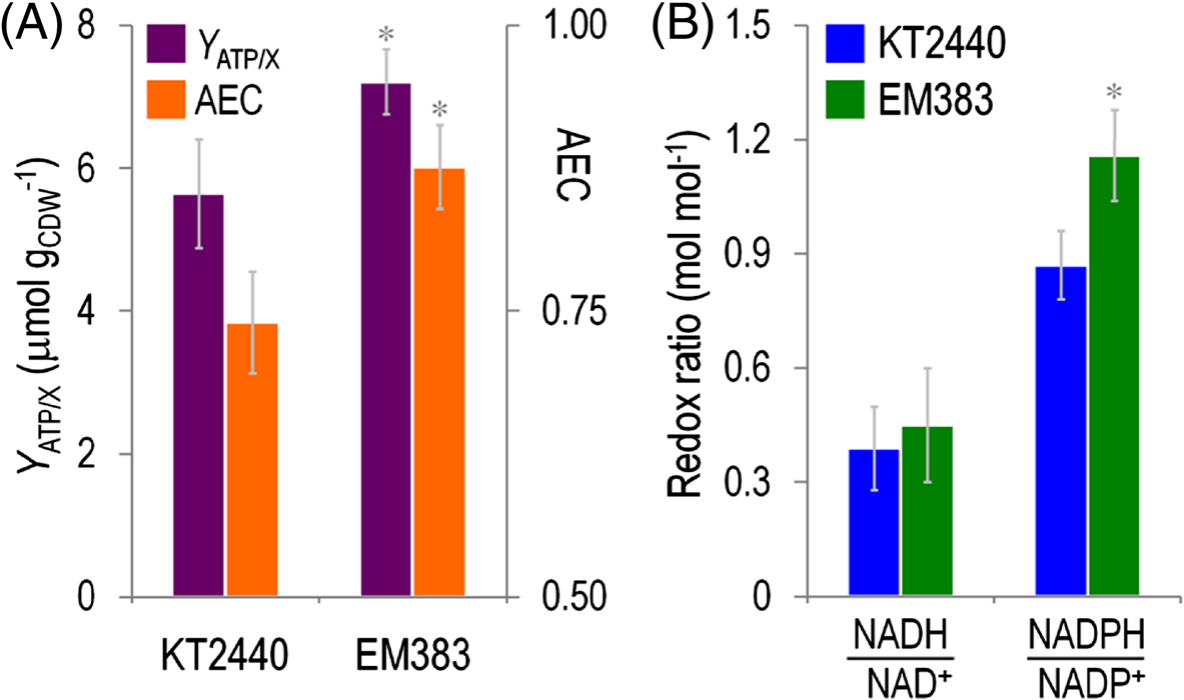
**Characterization of the energy and redox status of wild-type** ***P*** **.** ***putida*** **KT2440 and the streamlined strain EM383. (A)** The ATP content on biomass (*Y*_ATP/X_) and the adenylate energy charge (AEC) were calculated for exponentially-growing cells in shaken-flask cultures using M9 minimal medium containing 0.2% (w/v) glucose. Each bar represents the mean value and SD of the ATP content on biomass (CDW, cell dry weight) or the adenylate energy charge for duplicate measurements from at least three independent experiments. **(B)** Redox ratios were determined from the absolute intracellular concentrations of NAD^+^, NADH, NADP^+^, and NADPH. The pyridine nucleotide cofactors were enzymatically determined in exponentially-growing cells in shaken-flask cultures using M9 minimal medium containing 0.2% (w/v) glucose. Bars represent mean values along with SD of the corresponding parameter for duplicate measurements from at least three independent experiments. The asterisk (*) indicates a significant difference for strain EM383 as compared to wild-type KT2440 according to the Student’s *t* test (*P* <0.05).

The growth parameters described in the preceding section indicated that *P. putida* EM383 had an enhanced capacity to transform metabolic precursors into biomass. The biochemical reasons for this phenomenon were analyzed by exploring the energy and redox status of the cells at stake. Figure 4A shows the energy standing of the strains under examination in terms of both the ATP availability and the adenylate energy charge (AEC), a parameter which weights the ATP content against all the three possible phosphorylated forms of adenine. During exponential growth on glucose, EM383 cells had a 1.6-fold higher content of ATP per biomass unit [*Y*_ATP/X_, in μmol g cell dry weight (CDW)^−1^] than *P. putida* KT2440. This feature was translated into a 1.2-fold increase in the AEC. This surplus of high-energy phosphate in *P. putida* EM383 not only helps explaining the enhanced growth of this strain under different culture conditions, but also endows cells with an extra capacity to sustain extra ATP (and other NTPs) consuming functions. In close connection with the energy status, a significant increase in the NADPH/NADP^+^ ratio was observed in *P. putida* EM383 strain as compared to the wild-type (Figure 4B). It is remarkable that such redox charge was manifested mostly for anabolic processes, as the NADPH/NADP^+^ ratio was 1.3-fold higher in *P. putida* EM383 than in strain KT2440, but not in its catabolic counterpart (i.e., the NADH/NAD^+^ ratio, which remained more or less the same in the two strains). The larger split of NADPH signals this strain as more capable to sustain biotransformations that demand a higher redox charge [20].

The increased share of NADPH in the nicotinamide adenine pool does not justify *per se* the considerable increase in biomass yield reported in Figure 3. Therefore, our attention was turned to acetyl-coenzyme (CoA) as the central metabolic precursor for a large variety of cellular building blocks [70]. Not only does acetyl-CoA serve as the acyl carrier and donor for citrate synthesis within the tricarboxylic acid cycle (which ultimately results in ATP synthesis in the respiratory chain), but it also has important biogenic properties [72]. This molecule, also referred to as the *hub of metabolism*, is the precursor for the biosynthesis of fatty acids, various amino acids, *N*-acetylated compounds, and biopolymers such as polyhydroxyalkanoates. Measurement of its availability as a coarse descriptor of metabolic status was then carried out. When the concentration of this intermediate was assessed in *P. putida* EM383 grown in M9 medium with glucose as sole C source during the mid-exponential phase of growth, a 1.3-fold increase of the endogenous acetyl-CoA pool was observed as compared to that in *P. putida* KT2440 (134 ± 12 *vs*. 104 ± 9 nmol g_CDW_^−1^, respectively). These figures indicate that *P. putida* EM383 could deliver more acetyl-CoA to the synthesis of different metabolites which, by default, would be funnelled into biomass generation. The prediction is, however, that this same metabolic currency can be diverted to biosynthesis of other compounds of interest that demand a strong influx of acetyl-CoA in their corresponding pathways [70,72]. But how all these emerging biochemical properties translate into phenotypes of interest in a host of recombinant DNA?

### Dealing with endogenous redox stress

**Figure 5:**
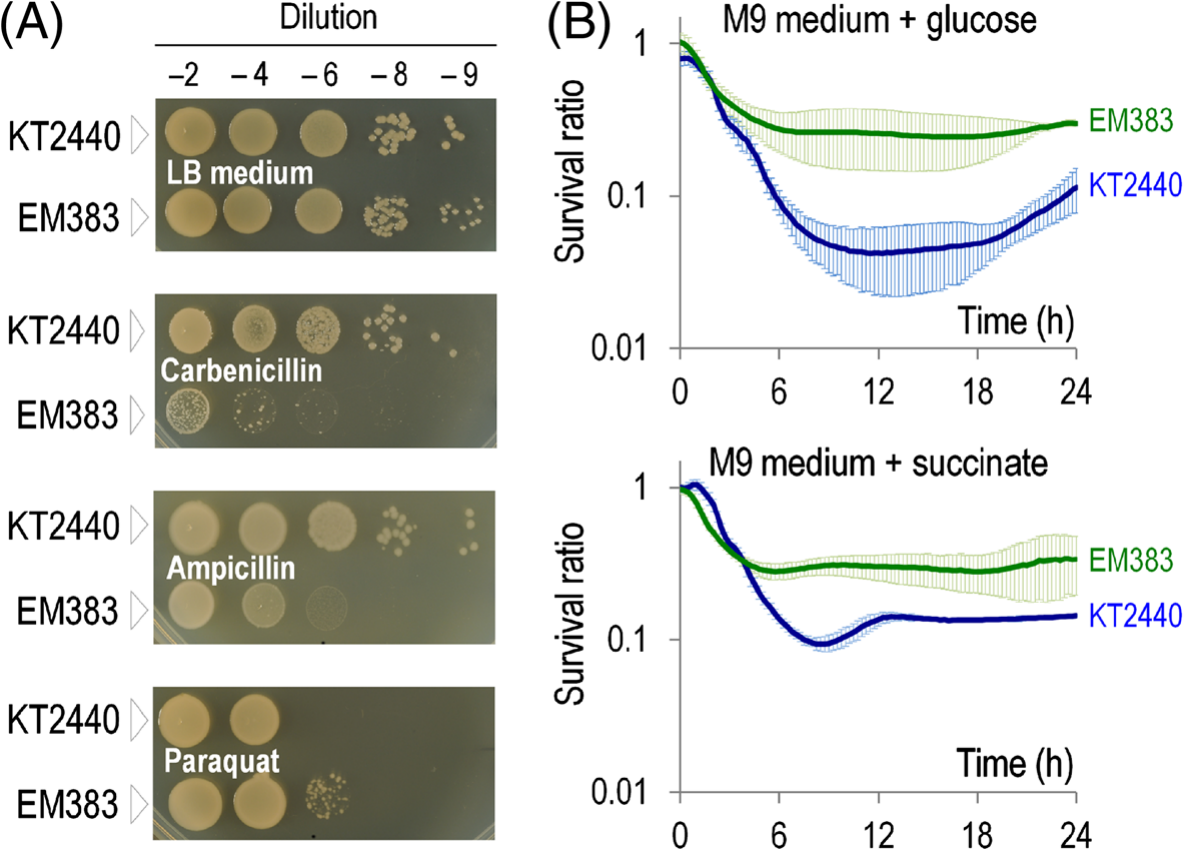
**Cell survival of wild-type** ***P*** **.** ***putida*** **KT2440 and the streamlined strain EM383 in the presence of different stressors. (A)** Drop assays were used to compare the fitness of wild-type KT2440 and strain EM383 when exposed to different chemical stressors. Overnight cultures were diluted in PBS and spotted onto LB agar plates supplemented with the particular compound (250 μg ml^−1^ carbenicillin, 50 μg ml^−1^ ampicillin, or 10 μM paraquat). LB medium was used in the control plate. **(B)** Survival ratio plots of wild-type KT2440 (blue) and strain EM383 (green) when exposed to 10 μM paraquat during 24 h. The survival ratio was calculated by dividing the optical density at 600 nm (OD_600_) of cultures with the drug to the OD_600_ of control cultures without paraquat along the time, thus merging the intrinsic effect of the stressor with the growth capability of each strain. Cells were grown in M9 minimal medium amended with 0.2% (w/v) of either glucose or succinate. The mean survival ratio values are plotted along with the SD of three independent experiments.

As mentioned above, one of the inherent qualities of *P. putida* is its ability to host redox reactions that often result in a high level of intracellular ROS [21]. One way to mimic such oxidative stress is treating cells with the chemical paraquat (1,1′-dimethyl-4,4′-bipyridinium dichloride). This compound is reduced *in vivo* by NADPH and then oxidized by an electron receptor such as dioxygen to produce superoxide, a major contributor to the ROS pool [73]. Therefore, paraquat both decreases NADPH and causes endogenous ROS (which itself has to be counteracted with enzymes that are ultimately fuelled by NADPH). The simple spot dilution test shown in Figure 5A indicated that *P. putida* EM383 performs better in the presence of the oxidative agent than the wild-type strain – a trait that correlates with the high NADPH/NADP^+^ ratio reported above. To have a better quantification of this phenomenon, the survival ratio of either strain was examined (Figure 5B) when grown in M9 minimal media with either glucose or succinate as the C source and in the presence of paraquat as detailed in the Methods section. The resulting curves expose both the early sensitivity to the stressor and how well they recover after the insult, since the dimensionless survival ratio merges both the tolerance and the intrinsic growth ability of each strain. Figure 5B clearly shows a better response of the cell-factory strain in both situations: after 8 h, the survival ratio of the streamlined strain EM383 in glucose was 0.26 ± 0,10 (0.05 ± 0.02 for the wild-type strain), while in succinate the survival ratio of strain EM383 was 0.30 ± 0.04 (0.10 ± 0.01 for the wild-type strain). This dataset documents that the observed increase of the redox charge of *P. putida* EM383 is concomitant with (and perhaps the reason of) the superior tolerance of this strain to endogenous redox stress and ROS damage. Figure 5A also shows that strain EM383 was more sensitive to the β-lactam antibiotics carbenicillin and ampicillin. This characteristic is surely originated in the lack of flagella, as observed before [57].

**Figure 6:**
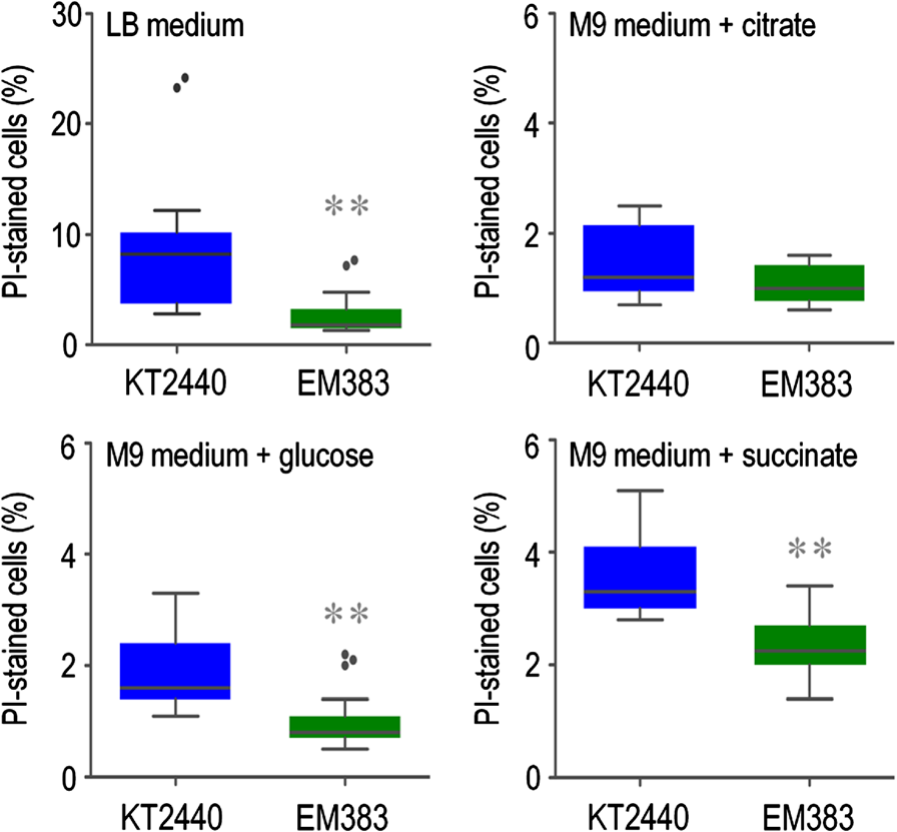
**Cellular viability assay.** The cellular viability of wild-type KT2440 (blue) and the streamlined strain EM383 (green) was compared in stationary-phase cultures. Cells grown overnight in LB medium or in M9 minimal medium added with 0.2% (w/v) of either citrate, glucose, or succinate were stained with propidium iodide (PI) and analysed by flow cytometry to estimate the percentage of dead cells (i.e., PI^+^ cells). The results of at least four biological independent experiments are represented as box plot charts (Tukey-style box plots). The median is marked as a grey line within the charts and the dark grey dots outside the box represent the data outliers. The results for *P. putida* KT2440 grown in M9 minimal medium with citrate and some of the replicas in LB and M9-glucose are taken from Martínez-García *et al*. [36]. The asterisks (**) indicate a significant difference for strain EM383 as compared to wild-type KT2440 according to the Mann–Whitney *U* test (*P* <0.01).

**Figure 7:**
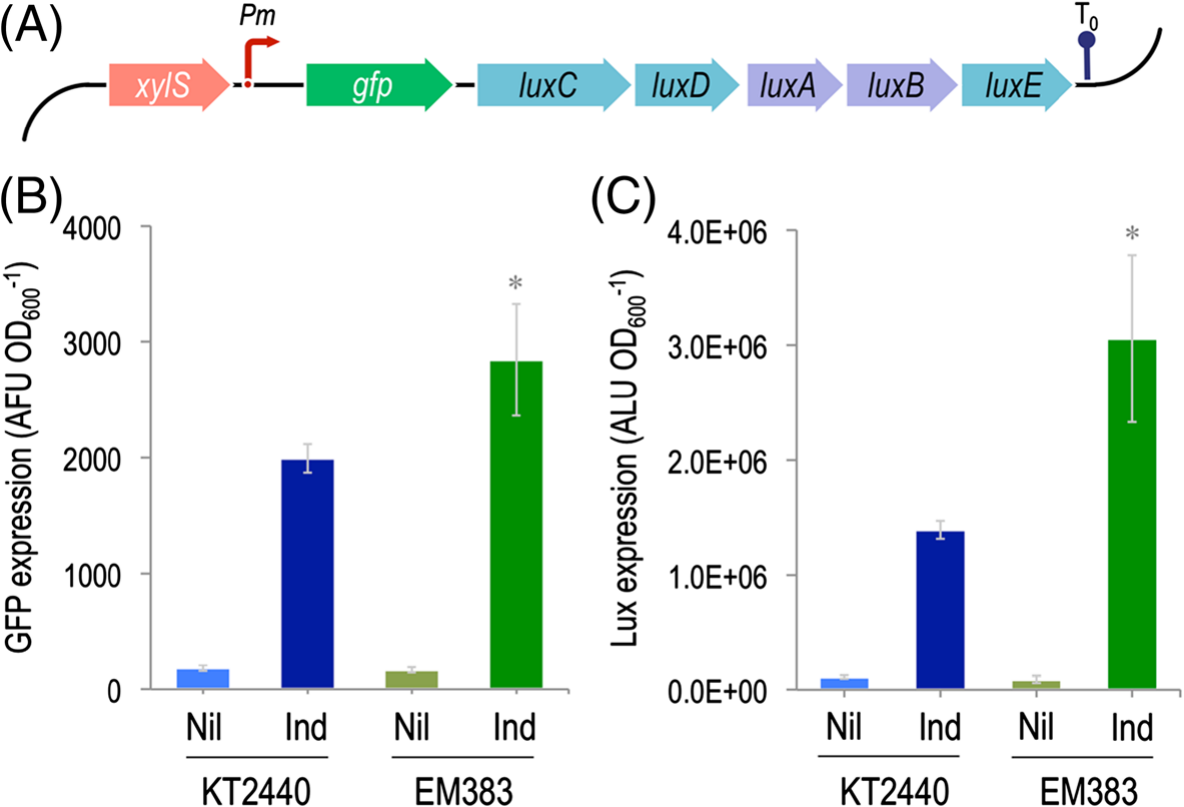
**Evaluation of** ***P*** **.** ***putida*** **EM383 as a** ***chassis*** **for the heterologous expression of** ***gfp*** **and** ***luxCDABE*** **. (A)** Schematic representation of the bi-cistronic GFP-LuxCDABE reporter in which both *gfp* (GFP: green fluorescent protein) and *luxCDABE* (LuxC: fatty acid reductase, LuxD: acyl transferase, LuxE: acyl-protein synthase, LuxAB: luciferase) from *Photorhabdus luminescens* are placed under the control of the inducible *Pm* promoter. The activity of *Pm* is controlled by the transcriptional regulator XylS. The transcriptional terminator included in the plasmid backbone is depicted as T_0_. The elements in this outline, borne by plasmid pGL-XP, are not drawn to scale. The reporter plasmid pGL-XP was used to establish a comparison of the expression levels of *gfp* **(B)** and *luxCDABE* **(C)** in wild-type KT2440 and in the streamlined EM383 strain in response to 3-methylbenzoate. Overnight cultures in rich LB medium were diluted to an optical density at 600 nm (OD_600_) of 0.1, cells were further grown for 2 h, and then induced with 1 mM 3-methylbenzoate for 24 h. The reporter expression level was calculated by dividing either the arbitrary fluorescence units (AFU) or the arbitrary luminescence units (ALU) by the OD_600_. The bars represent the media and SD of three measurements from biological triplicates. Nil, no inducer; Ind, induced. The asterisk (*) indicates a significant difference for strain EM383 as compared to wild-type KT2440 according to the Student’s *t* test (*P* <0.05).

Endogenous ROS stem not only from added-on redox reactions, but also from cell aging and nutrient starvation at the stationary phase [74]. The next obvious question was therefore whether *P. putida* EM383 can also deal better with such an inevitable physiological condition that all bacteria have to go through. To examine this issue propidium iodide was used along with cell cytometry as a reliable method to quantify cellular death, as this dye only stains bacteria with damaged membranes [75]. As shown in Figure 6, after overnight growth in LB medium, the cultures of the wild-type strain contained many more dead cells (8.2%) than in the *P. putida* EM383 counterpart (1.8%). A lower stationary-phase associated mortality of *P. putida* EM383 was also observed in M9 minimal medium with glucose or succinate, while no significant difference was observed when citrate was used as the C source (Figure 7). These results reflect the combination of the known effects of lacking flagella [57] and the prophage load [36], that in the present case seem to add to each other for increasing very significantly stationary phase survival.

### *P. putida* EM383 as a host of cloned DNA

Once the gains in physiological traits and oxidative stress resistance of *P. putida* EM383 were well characterized, a number of tests was run to validate the performance of this strain as a suitable acceptor and host of recombinant DNA. To this end, different parameters were considered that are often decisive for the adoption of a host of recombinant constructs. Paramount to this practical purpose is the acquisition of exogenous DNA through either electroporation or conjugation. First, the efficiency of *P. putida* EM383 as a receiver of plasmids through electroporation was tested. As an example, pSEVA251 [65], an standard, kanamycin resistant, broad-host-range cloning vector endowed with a promiscuous multi-copy RSF1010 origin of replication, was used. As shown in Table 3, the multi-deleted strain maintained its transformation capacity within the same order of magnitude known for the wild-type bacterium. This state of affairs changed dramatically when conjugation was used instead of electroporation as the method of choice. The mobilizable vector pSEVA221 [65] was adopted for this assay. This *oriT*^*+*^ plasmid is identical to the pSEVA251 vector described before, excepting that the origin of replication is that of the low-copy-number RK2 replicon, which is predicted to generate less noisy conjugal transfer events. The data shown in Table 3 revealed that *P. putida* EM383 had a significantly higher capacity of plasmid acquisition through conjugal delivery than the wild-type host. But once plasmids get in, what is their fate and their ease of recovering? To answer this question, *P. putida* EM383 and *P. putida* KT2440 were transformed with pGL-XP (Table 1, see next section for further details), a streptomycin and kanamycin resistant, broad-host-range plasmid that replicates through a multicopy *oriV* from plasmid pBBR1. Various transformants of each host were then lysed for extracting back the plasmid and inspect its quality. During the procedure, the washing step with a buffer containing a chaotropic agent, recommended by the mini-prep kit manufacturer when using *endA*^+^ strains, was eliminated to assess the possible effect of the two deleted DNases in the quality of plasmid DNA preparation (see the Methods section for details). As shown in Additional file 1: Figure S1, net plasmid recovery from *P. putida* EM383 was ~20% higher than that from the wild-type counterpart starting from the same amount of biomass. With these plasmid DNA preparations in hand, the DNA was digested with the restriction enzyme *Psh*AI and observed its digestion pattern on a 1% (w/w) agarose gel. As shown in Additional file 1: Figure S1, digestion of the plasmid purified from the wild-type strain (lanes 1 to 6) were prone to degradation, where those from the streamlined strain EM383 produced clearly-cut and non-degraded bands in the agarose gel (lanes 7 to 12). Taken together, the conclusion of all these experiments is that the streamlined strain EM383 does generally better than the parental *P. putida* bacteria as a holder of incoming DNA, the improvement being more apparent in the ease of conjugation, extraction of plasmids, and in the quality of the thereby recovered plasmid DNA.

**Table 3:** **Performance of the wild-type** ***P*** **.** ***putida*** **KT2440 and the streamlined strain EM383 as a host for recombinant DNA**

***P. putida strain***	**Plasmid transfer efficiency (transformants per 1 × 10** ^**9**^ **cells)**	
	**Mating** ^***a***^	**Electroporation** ^***b***^
KT2440	(5 ± 1) × 10^6^	5,483 ± 3,060
EM383	(18 ± 5) × 10^6^	1,642 ± 822

^*a*^The plasmid transfer efficiency by tri-parental mating was calculated using vector pSEVA221, and the figures represent the mean value and SD of three biological replicates.

^*b*^The plasmid transfer efficiency by electroporation was calculated using vector pSEVA251, and the figures represent the mean value and SD of three biological replicates when using 1 ng of vector.

### Physiological vitality of *P. putida* EM383 exposed with a bi-cistronic GFP-LuxCDABE reporter

In order to get a quantitative measure of the total enhancement of *P. putida* as a host of recombinant constructs the diagnostic plasmid pGL-XP was introduced in both the wild-type strain and in *P. putida* EM383 (Figure 7A). This is a construct based on vector pGLR1 [76], which merges every feature of the steps that goes from engineering a synthetic pathway to have it expressed in a heterologous host. Plasmid pGL-XP (which was used in the plasmid quality tests detailed above), consists of a bi-cistronic and co-transcribed operon formed by a leading GFP fluorescent reporter followed by luminescence-producing genes *luxCDABE* of *Photorhabdus luminescens.* The operon is transcribed through an expression system recruited from the pWW0 TOL plasmid of *P. putida* mt-2, consisting of the transcriptional factor XylS (which is activated upon exposure to 3-methylbenzoate, 3-*m*B) and its cognate target promoter *Pm* (Figure 7A). The readout of the two reporters thus embodies plasmid replication and stability, transcriptional activity, solvent tolerance (since 3-*m*B triggers a heat-shock like response in the cells [77]), protein production and folding (GFP), and tolerance to a high NADPH and ATP demand (both cofactors are needed for bioluminescence [22]) – all in the same lot. On this basis, cells of each strain were grown in LB medium, induced XylS with 1 mM 3-*m*B, and measured GFP and light emission after 24 h of incubation (Figure 7B). Note that, while GFP activity is cumulative (once folded, GFP-borne fluorescence remains in the cells), luminescence depends at all times on physiological availability of ATP and reducing power. The data of Figure 7B thus merges all the events that cells have gone through since the time of induction. Inspection of the results not only endorse the worth of *P. putida* EM383 as an expression host but also highlights the adequacy of this streamlined derivative to host reactions that consume both ATP and NADPH. If one takes luminiscence readout as a quantitative descriptor of combined physiological vigour and makes a correction by a possible effect in gene expression (as revealed by GFP), the benefit of deleting the gene set that is lacking in *P. putida* EM383 would increase physiological vitality by >50%.

## Conclusion

The data above emphasize the value of environmental bacteria such as *P. putida* KT2440 as the starting point of a platform strain with a degree of robustness and metabolic capacities that cannot be delivered by other bacterial hosts [21,26–29]. The judicious removal of just a limited number genes encoding undesirable traits has sufficed to upgrade the already useful properties of *P. putida* towards an optimal and standardized *chassis* for synthetic biology and metabolic engineering, especially in the cases where the pathways under study generate toxic intermediates and/or demand a biochemical background that supplies enough ATP and NADPH to sustain heterologous bioreactions. Note that the present approach does not focus on improving the genetic architecture of the implanted constructs, but it ensures the timely provision of metabolic currency that feeds the gene expression flow and helps counteracting ROS. Although EM383 strain (which is a *recA* mutant) was found to be superior to any other derivative of *P. putida* KT2440 as a host for heterologous gene expression, an isogenic *recA*^*+*^ counterpart (*P. putida* EM42, Table 1) is available as well – although we have seen no advantage in its use. Therefore strain *P. putida* EM383 can be seen as the first of a series of *P. putida* variants (*Pseudomonas* 2.0 and beyond) that will be increasingly refactored as one biochemical and genomic *chasses* of choice for holding harsh biotransformation reactions not feasible with current microbial platforms.

## Methods

### Bacterial strains, plasmids, culture media and growth conditions

Table 1 lists the bacterial strains and the plasmids used in this work. Bacteria were grown routinely in LB medium (10 g l^−1^ tryptone, 5 g l^−1^ yeast extract, and 5 g l^−1^ NaCl). M9 minimal medium [78], amended with different C sources at the concentrations indicated in the text, was also used for physiology experiments. *P. putida* was cultured at 30°C while *E. coli* cells were grown at 37°C. Antibiotics, when needed, were added at the following final concentrations: 150 μg ml^−1^ ampicillin for *E. coli* and 500 μg ml^−1^ for *P. putida*; 50 μg ml^−1^ kanamycin; 50 μg ml^−1^ streptomycin; and 250 μg ml^−1^ carbenicillin. Other supplements were added to the culture media during the deletion procedure (40 μg ml^−1^ 5-bromo-4-chloro-3-indolyl-β-D-galactopyranoside and 1 mM isopropyl-β-D-1-thiogalactopyranoside) [58]. The growth kinetic of the strains under examination was determined by following the OD_600_ of the cultures in 96-well microtiter plates using a SpectraMax M2e microplate reader (Molecular Devices, Sunnyvale, CA, USA). The final OD_600_ at 24 h (a proxy of substrate *vs.* biomass conversion) was measured in 50-ml Erlenmeyer flasks filled with 10 ml of culture medium grown with shaking at 170 rpm.

### DNA techniques, plasmid construction, and mating

DNA was manipulated using routine laboratory techniques [78]. Plasmid DNA was obtained using the QIAprep Spin Miniprep kit (Qiagen, Inc., Valencia, CA, USA). This kit includes a proprietary solution, termed buffer PB, containing a chaotropic agent (guanidine hydrochloride), which inactivates any endonuclease activity (the manufacturer recommends to use it when preparing plasmids from *E. coli endA*^+^ strains). To evaluate the impact of nucleases from the bacterial host on the quality of the plasmid DNA extracted, the wash step with buffer PB was omitted in some preparations. DNA amplified through the polymerase chain reaction (PCR) was purified with NucleoSpin Extract II (Macherey-Nagel, Düren, Germany). Oligonucleotides were purchased from Sigma-Aldrich Co. (St. Louis, MO, USA), and their sequences are indicated in Additional file 1: Table S2. Colony PCR was performed using a single colony from a fresh agar plate and transferred directly into the PCR reaction tube. All constructs were verified by DNA sequencing (Secugen SL, Madrid, Spain). Plasmid pSEVA221 (Table 1) was transferred from the donor *E. coli* DH5α into *P. putida* by tripartite mating, using *E. coli* HB101/pRK600 as the helper strain [79,80]. When needed, other plasmids were introduced into *P. putida* strains by electroporation [59]. Agarose gel analysis and densitometry were conducted using a Molecular Imager VersaDoc™ apparatus (Bio-Rad Corp., Hercules, CA, USA).

### Genomic editing

All the chromosomal deletions discussed in this work were done using the I-*Sce*I methodology [58,59], in which upstream (TS1) and downstream (TS2) segments of homologous DNA were separately amplified and then joined by means of splicing-by-overlap extension (SOEing) PCR [81]. The joined TS1-TS2 segments were digested with appropriate enzymes (Additional file 1: Table S1), cloned into the I-*Sce*I-bearing pEMG vector and then transferred to *P. putida* (carrying the I-*Sce*I expression plasmid pSW-I [64]) as described by Martínez-García and de Lorenzo [59]. Cointegrates were resolved by induction of I-*Sce*I expression with 15 mM 3-*m*B. Kanamycin-sensitive clones were next analyzed by PCR to verify the deletion at stake. Plasmid pSW-I was cured after growth without selective pressure and its loss confirmed by both sensitivity to 500 μg ml^−1^ ampicillin and colony PCR with the diagnostic oligonucleotides indicated in Additional file 1: Table S1.

### Determination of the AEC, ATP content on biomass, and redox ratios

AEC is a quantitative measure of the relative saturation of high-energy phospho-anhydride bonds available in the adenylate pool [82,83], following the formula:

$$ \mathrm{A}\mathrm{E}\mathrm{C}=\left(\left[\mathrm{A}\mathrm{T}\mathrm{P}\right]+0.5\left[\mathrm{A}\mathrm{D}\mathrm{P}\right]\right)/\left(\left[\mathrm{A}\mathrm{T}\mathrm{P}\right]+\left[\mathrm{A}\mathrm{D}\mathrm{P}\right]+\left[\mathrm{A}\mathrm{M}\mathrm{P}\right]\right) $$

The AEC values were calculated according to the individual ATP, ADP, and AMP content in deproteinized extracts obtained from batch cultures of the strains [84]. As a further measure of the energy status, the yield of ATP on biomass (*Y*_ATP/X_) was obtained by normalizing the molar ATP content to the CDW of culture samples. The *in vitro* nucleotide determinations were based on a commercially available ATP bioluminescence assay [24].

The redox status was explored through the assessment of the intracellular levels of pyridine nucleotide cofactors. The concentration of NAD^+^, NADH, NADP^+^, and NADPH was estimated using *in vitro* cyclic assays [57,85], starting with the rapid inactivation of the metabolism of exponentially-growing bacterial cells, followed by either acid or alkaline nucleotide extraction. Redox ratios were derived from these measurements. The intracellular concentration of the nucleotides, from which redox ratios were derived, was calculated based on previously described assumptions [24,86].

### Flow cytometry

Strains under examination were retrieved from frozen 20% (v/v) glycerol stocks and grown overnight at 30°C in either rich or minimal media. Samples of cells pre-grown in LB medium were diluted 1:5 in phosphate-buffered saline (8 mM Na_2_HPO_4_, 1.5 mM KH_2_PO_4_, 3 mM KCl, and 137 mM NaCl, pH =7.0), while bacteria pre-grown in M9 minimal medium were not diluted. In either case, cells were stained with propidium iodide at a final concentration of 1 μg ml^−1^, and analyzed in a Gallios™ flow cytometer (Beckman Coulter Inc., Pasadena, CA, USA) [57,84]. Samples were excited at 488 nm and fluorescence determined at 617 nm using a 620/30 nm band-pass filter. For the experiments, ≥100,000 events were counted per sample and the percentage of propidium iodide-retaining cells (i.e., dead bacteria) was determined.

### Endogenous oxidative stress tests

For a gross estimation of the toxicity of the oxidative stress of paraquat, cells under examination were pre-grown overnight in LB medium and serially diluted (10^−2^ to 10^−9^) in phosphate-buffered saline. A 10-μl sample of each dilution was laid onto the agar surface of Petri dishes with LB agar medium added or not with 10 μM of the stressor, incubated for 24 h at 30°C, and photographed. Stress resistance in liquid cultures was assessed by diluting the same overnight cultures to an OD_600_ of 0.05 in M9 medium with either 0.2% (w/v) succinate or glucose as the sole C source and supplemented or not with 10 μM paraquat. OD_600_ was then followed for the next 24 h. The survival ratio was calculated as the OD_600_ of cultures added with paraquat and normalized to the OD_600_ of cultures without the stressor along time [57]. The dimensionless ratio OD_600_(+paraquat)/OD_600_(no stressor) thus merges the tolerance to paraquat along with the growth capacity of each strain.

### Heterologous gene expression assays

The activity of the bi-cistronic reporter GFP-LuxCDABE borne by plasmid pGL-XP (Table 1), which is inducible by 3-*m*B, was used as an indicator of heterologous gene expression in the *P. putida* strains under examination. Bacteria bearing the reporter construct were grown overnight in LB medium with streptomycin to ensure plasmid retention. Samples were then diluted to an OD_600_ of 0.1 in fresh LB medium, further grown for 2 h, and the *Pm* promoter was induced with 1 mM 3-*m*B. Samples were taken at different time intervals, and fluorescence and bioluminescence measured simultaneously. For these assays, 200-μl aliquots were placed on 96-well microtiter plates (Costar black plates with clear bottom; Thermo Fisher Scientific Inc., Pittsburgh, PA, USA), and fluorescence was measured in a SpectraMax M2e (Molecular Devices, LLC, Sunnyvale, CA, USA), while bioluminescence was quantified in a Wallac Victor 2 (Perkin Elmer Corp., Waltham, Massachusetts, USA). The raw GFP and luminescence values were normalized by dividing signal output in either case by the OD_600_ of the culture at the harvesting time.

### Other analytical procedures

Succinate concentration was determined using an assay kit from Megazyme International Ireland (Wicklow, Ireland). Tests were done according to the manufacturer’s protocol through a coupled enzymatic assay and adjusting the concentrations of the reagents to a final volume of 1 ml. Glucose was measured in culture supernatants using another kit from R-Biopharm AG (Darmstadt, Germany), as per the manufacturer’s instructions. In either case, control mock assays were made by spiking M9 minimal medium with different amounts of the C sources under examination. Biomass yields (CDW measurements), specific growth rates, and C consumption during exponential growth were calculated from growth parameters in each culture condition as described elsewhere [24,57,69,84–87]. The acetyl-CoA content was determined by liquid chromatography coupled to mass spectrometry as previously detailed [88].

### Statistical analysis

All reported experiments were independently repeated at least twice (as indicated in the corresponding figure legend). The statistical significance between multiple comparisons was estimated by means of the Student’s *t* test at confidence levels of 95% or 99%. For the flow cytometry experiments, the median value is reported in box plots with the first and third quartiles. In these experiments, the statistical significance was analyzed with the Mann–Whitney *U* test.

## Additional file

Additional file 1: Table S1. Genomic coordinates of the eleven deletions introduced in *Pseudomonas putida* KT2440 to construct the streamlined strain EM383. **Table S2.** Oligonucleotides used in this study. **Figure S1.** Evaluation of the streamlined strain as a host for heterologous plasmid DNA.

## Abbreviations

ATP: Adenosine triphosphate
ADP: Adenosine diphosphate
AMP: Adenosine monophosphate
NTPs: Nucleoside triphosphates
NADH: Nicotinamide adenine dinucleotide, reduced form
NADPH: Nicotinamide adenine dinucleotide phosphate, reduced form
OD_600_: Optical density measured at 600 nm
ORF: Open reading frame
rpm: Revolutions per minute
SD: Standard deviation
CDW: Cell dry weight
3-*m*B: 3-methylbenzoate
ROS: Reactive oxygen species
