# Supplementary material for: Pseudomonas 2.0: genetic upgrading of P. putida KT2440 as an enhanced host for heterologous gene expression
Source: Microb Cell Fact. 2014 Nov 11;13:159. doi: 10.1186/s12934-014-0159-3 (PMC4230525; doi:10.1186/s12934-014-0159-3)
Supplement: Additional file 1: Table S1. — Genomic coordinates of the eleven deletions introduced in Pseudomonas putida KT2440 to construct the streamlined strain EM383. Table S2. Oligonucleotides used in this study. Figure S1. Evaluation of the streamlined strain as a host for heterologous plasmid DNA. [file 12934_2014_159_MOESM1_ESM.pdf]

ADDITIONAL FILE 1

Table S1. Genomic coordinates of the eleven deletions introduced in *Pseudomonas putida* KT2440 to construct the streamlined strain EM383.

Deletion[a]	Gene or gene cluster	Number of genes targeted	Coordinates deleted (bp)		Extension of the deletion (bp)
			Start	End	
1	Prophage 1	72	4,372,649	4,427,414	54,766
2	Prophage 4	41	1,738,082	1,778,140	40,059
3	Prophage 3	32	2,586,633	2,625,819	39,187
4	Prophage 2	55	3,412,988	3,448,599	35,612
5	Tn7 transposase	4	6,161,914	6,168,509	6,596
6	*endA-1*	1	2,798,042	2,798,734	693
7	*endA-2*	1	3,819,907	3,820,872	966
8	*hsdRMS*	3	5,393,057	5,398,717	5,661
9	Flagellum	69	4,919,154	4,988,328	69,175
10	Tn*4652*	21	3,366,692	3,382,468	15,777
11	*recA*	1	1,827,143	1,828,210	1,068

[a] The deletion number identifies the order in which the specific gene or gene cluster was deleted. The information regarding genomic coordinates of each locus was derived from the reported sequence of *P. putida* KT2440 [1].

Table S2. Oligonucleotides used in this study.

Name	Sequence (5' → 3')[a]	Use
TS1(*endA1*)*Xma*I-F	TCCC**CCCGGG**GCTTGTGTCAGGCGCCGTTC	Deletion of *endA-1*
TS1(*endA1*)-R	*CCGAAGGAAGATTGACTGCGC*TCCTCAGGCCA GCGTTTGTAAG	Deletion of *endA-1*
TS2(*endA1*)-F	GCGCAGTCAATCTTCCTTCGG	Deletion of *endA-1*
TS2(*endA1*)*Bam*HI-R	CG**GGATCC**CAGCAAAGAGCTGCAGCGGATC TTC	Deletion of *endA-1*
TS1(*endA2*)*Eco*RI-F	CG**GAATTC**CGGGGGTTGAGCATTACCCGCTT	Deletion of *endA-2*
TS1(*endA2*)-R	*GGCCGCGCAGCCTTTGAAACGG*GGGGAAAAC ATATTTCAGGTTG	Deletion of *endA-2*
TS2(*endA2*)-F	CCGTTTCAAAGGCTGCGCGGCC	Deletion of *endA-2*
TS2(*endA2*)*Bam*HI-R	CG**GGATCC**GCATGCGCGTGCTGTATCACAGC	Deletion of *endA-2*
TS1(*hsdRMS*)*Eco*RI-F	GC**GAATTC**GTTCGTCGACTGGATGGAGC	Deletion of *hsdRMS*
TS1(*hsdRMS*)-R	*TCAGGCGCATAACGAATTTC*TGCTGGCTATGT CCCTATGG	Deletion of *hsdRMS*
TS2(*hsdRMS*)-F	GAAATTCGTTATGCGCCTGA	Deletion of *hsdRMS*
TS2(*hsdRMS*)*Bam*HI-R	CG**GGATCC**TTGTCTGGCTTTTGCATCAG	Deletion of *hsdRMS*
TS1(Tn*4652*)*Xma*I-F	TCCC**CCCGGG**CCGTTCTGGCCAATGCC GGGCA	Deletion of Tn*4652*
TS1(Tn*4652*)-R	*GTCCATTTTGCCGTGCCCTGCTG*CCCCCAAGC GAGGCAAAAGC	Deletion of Tn*4652*
TS2(Tn*4652*)-F	CAGCAGGGCACGGCAAAATGGAC	Deletion of Tn*4652*
TS2(Tn*4652*)*Bam*HI-R	CG**GGATCC**ATGAAGGAATTCGTTTGGTGCAAG	Deletion of Tn*4652*
TS1(Tn*7*)*Eco*RI-F	CG**GAATTC**CCAGAACGGGTGCTAGCTGC	Deletion of Tn*7*
TS1(Tn*7*)-R	GTCCAGCTTGGCTTTCAAATCAG	Deletion of Tn*7*
TS2(Tn*7*)-F	*CTGATTTGAAAGCCAAGCTGGAC*ATTTTTAACG ATTGACTGTTAC	Deletion of Tn*7*
TS2(Tn*7*)-*Xma*I-R	TCCC**CCCGGG**CTCCGAGACCGCCGACCAAGA	Deletion of Tn*7*
3864F	ACTTGTACACCCGTGGTTCG	Diagnose deletion of prophage 1[b]
3864R	TGAACCAGCGTTCGATACTG	Diagnose deletion of prophage 1[b]
3065F	GTCGACGAGGTGGAATTGAG	Diagnose deletion of prophage 2[b]
3065R	GCAGAGGTTTTGTTGGGGTA	Diagnose deletion of prophage 2[b]
2277F	GCAGATCGAGGACTTCAAGC	Diagnose deletion of prophage 3[b]
2277R	GCACTCCATAGCACCCTAGC	Diagnose deletion of prophage 3[b]
1565F	CTGACCGAGGATCAGATGGT	Diagnose deletion of prophage 4[b]
1565R	CCGGGTTGAACTTCACGTAG	Diagnose deletion of prophage 4[b]
5406F	CATCTCCTTTCCAACCCAGA	Diagnose deletion of Tn*7*
5406R	CGTGCATACCAAACAACAGG	Diagnose deletion of

		Tn7
4335F	TACCGAGGAACACGAAAACC	Diagnose deletion of the flagellum[c]
4335R	TTGGCAGGTTGTCAGTGAAG	Diagnose deletion of the flagellum[c]
4741F	CTCGACCAGTCACCGAATTT	Diagnose deletion of *hsdRMS*
4741R	ACGATGGTGTGCAGGTTACA	Diagnose deletion of *hsdRMS*
2968F	GTGCTCCGATAGCGGCGGCAGCA	Diagnose deletion of Tn*4652*
2968R	GGGCGCATCGAACTGTCGATCTTC	Diagnose deletion of Tn*4652*
endA1-F	CGCTTTTCGCAGCAGCCTGCCTG	Diagnose deletion of *endA-1*
endA1-R	GAAGTAGGTGCGGGCGATCATGCC	Diagnose deletion of *endA-1*
endA2-F	CAATGCCGGCGAATACGGCCAAT	Diagnose deletion of *endA-2*
endA2-R	CAGGCCATCAGCAGCTGTTGCTG	Diagnose deletion of *endA-2*
recA-F	CTACGGCCCGGAATCGTCGGGTA	Diagnose deletion of *recA*
recA-R	GACCGCGCCGGTACGGCGGATGT	Diagnose deletion of *recA*
TS1(*recA*)*Eco*RI-F	CG**GAATTC**CGGTGGTGGCATTGCCGAAGC	Diagnose deletion of *recA* (TS1-TS2)[b]
TS2(*recA*)*Bam*HI-R	CG**GGATCC**TTCGAGCTTCAATAATCGTCG	Diagnose deletion of *recA* (TS1-TS2)[b]
endA-1-junction-F	CGTGAAGAAGCGCAGGCGCGC	Sequence boundaries of *endA-1* deletion
endA-1-junction-R	GTAATGCCGAACAGCCGGATGA	Sequence boundaries of *endA-1* deletion
endA-2-junction-F	CCGCTGAGTTTCAGCCTGGC	Sequence boundaries of *endA-2* deletion
endA-2-junction-R	GGCAGGCCAACCTGGGGCCGGCG	Sequence boundaries of *endA-2* deletion
Tn*4652*-junction-F	GTCTCGCGCCTTTATCCTTCA	Sequence boundaries of Tn*4652* deletion
Tn*4652*-junction-R	GTGACCGGCGCCACATAGAGGC	Sequence boundaries of Tn*4652* deletion
hsdRMS-junction-F	AATACTATAGGTTCAGTTCCC	Sequence boundaries of *hsdRMS* deletion
hsdRMS-junction-R	CTTAAGTTCAAGTTTTACAACG	Sequence boundaries of *hsdRMS* deletion
Tn*7*-junction-F	CCGACCTGGGAAGGTCGACTTT	Sequence boundaries of Tn*7* deletion
Tn*7*-junction-R	GATGACTTCCTAGGCCATTACTTA	Sequence boundaries of Tn*7* deletion

recA-junction-F	CTCAAGGAGCACCCACATGG	Sequence boundaries of *recA* deletion
recA-junction-R	GCTACCGCATTCGCGGGTGA	Sequence boundaries of *recA* deletion
Junction-Flagella-F	CGCCAAGCCTCGCTACCCGGCCTGCT	Sequence boundaries of flagellum deletion[c]
Junction-Flagella-R	CAGTTGATTCTGGTGGTGCACCCG	Sequence boundaries of flagellum deletion[c]
pEMG-F1	CCATTCAGGCTGCGCAACTGTTG	Sequence TS1-TS2 in pEMG[d]
pEMG-R1	CTTTACACTTTATGCTTCCGGC	Sequence TS1-TS2 in pEMG[d]
pSW-F	GGACGCTTCGCTGAAAACTA	Diagnose curation of the plasmid pSW-I[d]
pSW-R	AACGTCGTGACTGGGAAAAC	Diagnose curation of the plasmid pSW-I[d]

[a] Recognition site for the restriction enzymes specified are indicated in boldface in the DNA sequence, and complementary sequences used in splicing by overlap extension (SOEing) PCR amplifications are shown in italics.
[b] Taken from Martínez-García *et al*. [2].
[c] Taken from Martínez-García *et al*. [3].
[d] Taken from Martínez-García and de Lorenzo [4].

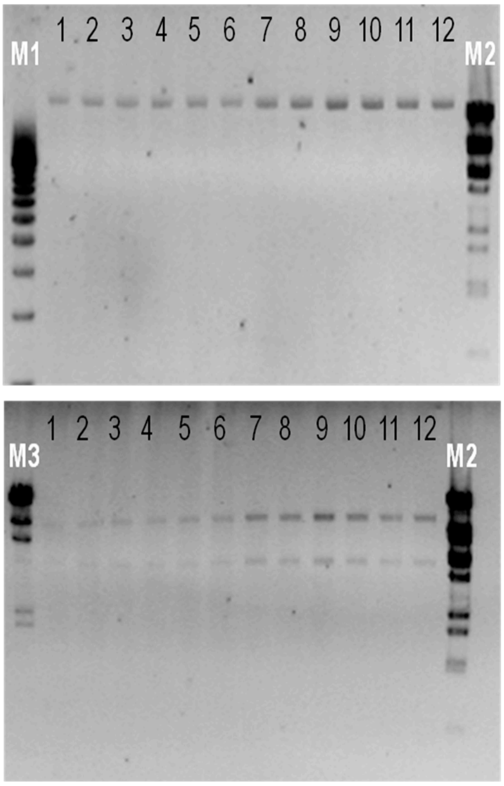

Figure S1. Evaluation of the streamlined strain as a host for heterologous plasmid DNA. Top panel: Gel electrophoresis analysis of six independent plasmid DNA purifications from saturated LB cultures of either the wild-type *P. putida* strain KT2440 (lanes 1 to 6) or *P. putida* strain EM383 (lanes 7 to 12) run on an 1% (w/v) agarose gel and visualized under ultraviolet light after staining with ethidium bromide. **Bottom panel:** Gel electrophoresis analysis of the *Psh*AI-digested plasmids obtained from the wild-type strain KT2440 (lanes 1 to 6) or strain EM383 (lanes 7 to 12) as explained above, and run on an 1% (w/v) agarose gel. Flanking lanes in both gels correspond to different DNA size markers [M1, 500-bp Molecular Ruler *EZ* Load™ (Bio-Rad, Berkeley, CA, USA); M2, λ DNA digested with *Bst*EII; and M3, λ DNA digested with *Hin*dIII]. Plasmid DNA purification and analysis was conducted following routinely procedures as described elsewhere [5].

References

1. Winsor GL, Lam DK, Fleming L, Lo R, Whiteside MD, Yu NY, Hancock RE, Brinkman FS: ***Pseudomonas* Genome Database: improved comparative analysis and population genomics capability for *Pseudomonas* genomes.** *Nucleic Acids Res* 2011, **39:**D596-D600.
2. Martínez-García E, Jatsenko T, Kivisaar M, de Lorenzo V: **Freeing *Pseudomonas putida* KT2440 of its proviral load strengthens endurance to environmental stresses.** *Environ Microbiol* 2014, **In press,** DOI: 10.1111/1462-2920.12492.
3. Martínez-García E, Nikel PI, Chavarría M, de Lorenzo V: **The metabolic cost of flagellar motion in *Pseudomonas putida* KT2440.** *Environ Microbiol* 2014, **16:**291-303.
4. Martínez-García E, de Lorenzo V: **Engineering multiple genomic deletions in Gram-negative bacteria: analysis of the multi-resistant antibiotic profile of *Pseudomonas putida* KT2440.** *Environ Microbiol* 2011, **13:**2702-2716.
5. Sambrook J, Maniatis T, Fritsch EF: *Molecular cloning: A laboratory manual.* Cold Spring Harbor, N.Y.: Cold Spring Harbor Laboratory Press; 1989.
